# Seasonality of tuberculosis in the Republic of Korea, 2006-2016

**DOI:** 10.4178/epih.e2018051

**Published:** 2018-10-20

**Authors:** Eun Hee Kim, Jong-Myon Bae

**Affiliations:** Department of Preventive Medicine, Jeju National University School of Medicine, Jeju, Korea

**Keywords:** Tuberculosis, Periodicity, Disease management, Vitamin D, Prevention

## Abstract

**OBJECTIVES:**

While the seasonality of notified tuberculosis has been identified in several populations, there is not a descriptive epidemiological study on the seasonality of tuberculosis in Korea. This study aimed to evaluate the seasonality of tuberculosis in Korea from 2006 to 2016.

**METHODS:**

Data regarding notified cases of tuberculosis by year and month was obtained from the *Infectious Diseases Surveillance* Yearbook, *2017* published by the Korea Centers for Disease Control and Prevention. Seasonal decomposition was conducted using the method of structural model of time series analysis with simple moving averages.

**RESULTS:**

While the trough season was winter from 2006 to 2016, the peak season was summer between 2006 and 2012, but shifted to spring between 2013 and 2016.

**CONCLUSIONS:**

Notified tuberculosis in Korea also showed seasonality. It is necessary to evaluate factors related to the seasonality of tuberculosis for controlling tuberculosis.

## INTRODUCTION

While the incidence rate of tuberculosis in Korea is ranked number one among the Organization for Economic Cooperation and Development countries, tuberculosis has the highest number of incident cases after chickenpox among infectious diseases under surveillance in Korea [1]. To eradicate tuberculosis, the government has constructed a national tuberculosis management system centered on health centers across the country since 1962 to conduct investigations [2]. Since 2000, a Web-based notification system centered on medical institutions was adopted to enable a more systematic approach to tuberculosis management [3]. The Korea Centers for Disease Control and Prevention (KCDC) has been publishing tuberculosis surveillance yearbooks of data collected like this for the planning, implementation, and evaluation of the national tuberculosis management policies [1]. However, the incidence rate of tuberculosis is still high in 2018. Thus, it is necessary to actively conduct a descriptive study of the epidemiological characteristics of tuberculosis patients in Korea to be used to establish a strategy for tuberculosis management.

The most noteworthy epidemiological characteristic of tuberculosis is its seasonality [4]. Related studies have reported that tuberculosis occurs mostly in the spring or summer and least in the fall and winter worldwide (Table 1) [5-19]. Given that many countries with various tuberculosis notification methods show the same seasonality, the most persuasive hypothesis is the effect of vitamin D concentration depending on the amount of sunshine in each season [20]. Therefore, some systematic reviews for evaluating the relationship between vitamin D deficiency and tuberculosis risk via a systematic review were published [21,22].

A nationwide survey of investigating blood vitamin D concentrations among adolescent children between the ages of 12 and 18 years in Korea showed that 90.5% of subjects were deficient at less than 20 ng/mL during the winter [23]. Thus, tuberculosis in Korea can be predicted to be related to seasonality as well. However, descriptive epidemiological studies on the seasonality of tuberculosis in Korea were unavailable as of September 2018. The aim of this study was to examine the seasonality of tuberculosis in Korea.

## MATERIALS AND METHODS

The number of patients notified for each year and month needed for analysis of seasonality of tuberculosis was obtained from the 2017 *Infectious Diseases Surveillance* Yearbook [1]. This database was compiled from tuberculosis patients or suspected tuberculosis patients based on the statistical information notified as new patients (initial treatment receivers) through the combined tuberculosis information management system in health centers and medical facilities nationwide. Therefore, recipients of re-treatment and patients with unclear previous treatment were not included. Tuberculosis cases were defined as those who showed clinical characteristics upon entry of the tuberculosis bacterium into the body and who tested positive to the tuberculosis bacillus. Suspected tuberculosis cases fit the diagnosis of tuberculosis based on the clinical, radiological, or histological observations but did not test positive to the tuberculosis bacillus. Both cases are subject to be reported.

The KCDC has adopted a Web-based notification system since 2000 and has been providing statistics on new patients for each month and year since 2001 [2]. However, there was some variance in the number of notifications with September 2005 seeing 5,284 notifications while the same month saw 2,301 notifications in 2004 and 2,860 notifications in 2006 [1]. For the stability of statistical data to be analyzed, the number of notifications per month for 10 years between January 1, 2006, and December 31, 2016, was obtained. To evaluate seasonality, the variable that represented season was created by dividing the year into 3-month periods: spring (Q1, March to May), summer (Q2, June to August), fall (Q3, September to November), and winter (Q4, December to February). Furthermore, as gender- and age-based information could not be found within the notified cases, the report rate per season per year was contraposed with a crude rate that applied to the population of registered citizens for each year.

To examine seasonality, seasonal decomposition among time series analysis methods was used to calculate the seasonal index [24]. For the models of seasonal decomposition time series models, there are multiplicative approaches and additive approaches. The multiplicative approach posits that the variance in the results can be explained by the product of the trend factor, circular factor, seasonal factor, and error. The additive approach posits that the variance can be explained by the sum of the four factors. Of these two models, the additive seasonal approach is taken if the seasonal variation is consistent despite the increase in time series, while the multiplicative seasonal approach is taken if seasonal variance increases or decreases depending on the increase or decrease of the time series [25]. Because the data of the current study had consistent seasonal variation across the flow of time, the additive approach was used. Moreover, the method of simple moving averages was used so that the width of the data for each period was equal to the number of weeks and was added identically at all time points in order to adjust for seasonal variation. All statistical analyses were conducted using SPSS version 18.0 (IBM Corp., Armonk, NY, USA). The present study was exempted from institutional review board because the data were anonymized.

## RESULTS

A total of 389,511 tuberculosis cases were notified nationwide from January 1, 2006, to December 31, 2016. Seasonally, summer was the peak season at 106,304 cases and winter was the trough season at 87,881 (Table 2). Among the months, July had the highest average number of notified cases while February had the lowest (Table 3).

**Figure 1.**
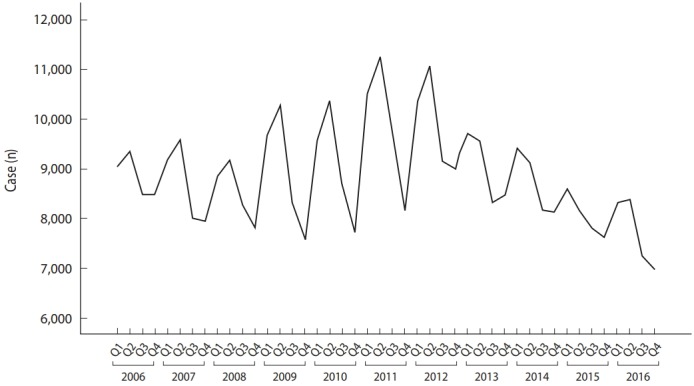
Seasonal notified tuberculosis cases, 2006-2016.

**Figure 2.**
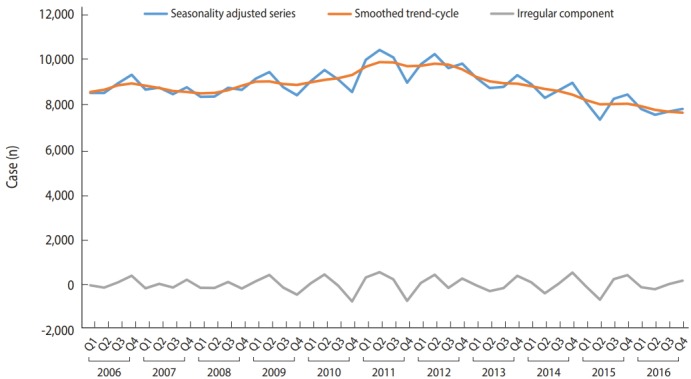
Time series decompositions of tuberculosis cases.

Looking at the trends in notified cases per season per year, there was a tendency for summers to be peaks and winters to be troughs from 2006 to 2012 and then it changed to a tendency for springs to be the peaks after 2013 (Figure 1). Figure 2 displays the results of seasonal decomposition of tuberculosis notifications from 2006 to 2016. Considering that the peak of season-adjusted series with seasonal factor removed surpassed the trend and circular series, it was shown that the reported rates change with sensitivity to seasonal changes.

Furthermore, we found the presence of a seasonally-based trend change in addition to the reduction in overall notified cases as shown in Table 4. The seasonal numbers in Table 4 showed that summer was the peak season at 813.315 followed by spring at 508.140, fall at -474.069, and winter at -847.385. However, analyzing after dividing the data into the 2006-2012 period and 2013-2016 period based on the trend change since 2013 showed that while 2006-2012 had the same order, 2013-2016 had a different order with spring at 469.568, summer at 318.068, winter at -346.932, and fall at -440.703.

## DISCUSSION

We confirmed that tuberculosis notifications in Korea have seasonality with summer being the peak season and winter being the trough season with these results. This was in line with the finding from a recent systematic review of seasonality in tuberculosis [26] that the prevalence of tuberculosis is high in the spring or summer and low in the fall or winter in 49 out of 57 studies.

While the amount of sunshine, changes in the temperature, indoor activity, and changes in immunity have been suggested as factors for seasonal change in tuberculosis, the hypothesis that the prevalence of tuberculosis is affected by the amount of sunshine depending on the latitude or season is the most powerful [27]. The fact that countries in the southern hemisphere show seasonality effects that are the opposite of those seen in countries in the northern hemisphere supports this [26]. Furthermore, a complex relationship can be inferred among factors such as an increase in indoor activities during winter leading to crowded living, and decreased the amount of sunshine leading to vitamin D deficiency, which in turn leads to decreased immunity and increased vulnerability to infections [28].

However, unlike the characteristics of most respiratory infectious diseases that occur more frequently in the winter, tuberculosis notifications increase in the spring and summer; two reasons have been suggested for this [29]. The first reason is that respiratory diseases that occur in the winter induce tuberculosis. Some studies conducted in South Africa on the seasonality of influenza, invasive pneumococcal disease, and tuberculosis showed that the incidence of invasive pneumococcal disease and tuberculosis reaches the peak about 3-4 months after the incidence of influenza reaches its peak [30-33]. The second reason is the length of disease progression for tuberculosis. The tuberculosis bacillus takes a long time to cause symptoms after entering the body. It takes the tuberculosis bacillus about 3-8 weeks to cause the tuberculin reaction after entering the alveoli. It takes about 3 months for symptoms to arise. This time may be delayed by up to 2 years depending on individual differences [34]. Looking at the results of our study that analyzed data based on tuberculosis report date reflecting these tuberculosis disease characteristics, it can be inferred that the infections occurred from winter to spring and notifications occurred from spring to summer.

One thing to note in the results of our study is that with the reduction in notified cases since 2013, the peak of incidence changed from summer to spring. However, we can infer that this may be due to changes in the national policy or notification system rather than changes in the epidemiological characteristics of tuberculosis from the fact that only the peak showed a change and the tendency for the winter to be the trough season did not change. In other words, the results may be explained by the active identification of tuberculosis patients due to the strengthening of the national tuberculosis management policies which then resulted in a decrease in the time between identification and notification. The KCDC has been strengthening patient management to select high-risk groups and actively conducting tests to identify patients early and lead to treatment based on the “First National Guideline for Tuberculosis Control (2013-2017)” established in 2013 [2]. It can be inferred that these efforts led to reductions in the number of tuberculosis notifications and in the time until notification.

The limitations of the current study are as follows. First, only the number of new tuberculosis patients was used and the basic characteristics of Koreans such as gender and age could not be identified. The data also included foreigners and suspected patients, so the research subjects could not be specified. However, nationality and the amount of sunshine are unrelated; also, considering the number of notifications in the scale of tens of thousands, we expect that the inclusion of foreigners and suspected patients would not cause disruptions in the seasonality variances. Further descriptive epidemiological studies aligned with the demographic characteristics of patients are needed. Second, the nature of tuberculosis makes it difficult to know the exact time of occurrence of tuberculosis based only on annual statistics. This is due to the long incubation period of tuberculosis. However, we took advantage of the fact that the data were annual statistics on first-diagnosis patients to assume that the time of tuberculosis report is the time of incidence. Third, our study was a descriptive epidemiological study, so further research on the causes of seasonality in tuberculosis notifications among Koreans is needed. An analytic epidemiological research that investigates vitamin D concentration level depending on the length of time of indoor activity for each age and gender group and finds its relationship with tuberculosis incidence is needed.

Understanding the seasonality of tuberculosis is very important in order to effectively and efficiently prevent it [27]. This is because concentrated prevention plans can be established in the season with high incidence by identifying and blocking or revising factors that are inferred to affect tuberculosis among differences between seasons. Furthermore, it is also helpful in that it can contribute to the advancement of the etiological knowledge about tuberculosis [17] and strengthen the surveillance mechanism [35].

## Figures and Tables

**Table 1.** Summaries of the seasonality of tuberculosis by countries

Areas	Country [reference]	Study period	Season (month)	
			Peak	Trough
Asia	Japan [5]	1998, 2000-2003	Spring-summer (May-Aug)	Fall-winter (Nov-Jan)
	China [6]	2005-2014	Spring (Mar)	Fall (Oct)
	Hong Kong [7]	1991-2002	Summer (Jul and Aug)	Winter (Jan and Feb)
	India [8]	1996-2001	Spring (Apr-Jun)	Fall-winter (Oct-Dec)
	Pakistan [9]	2006-2013	Spring (Apr-Jun)	Fall-winter (Oct-Dec)
	Kuwait [10]	1997-2006	Spring-summer (Mar-Aug)	Fall-winter (Aug-Dec)
	Iran [11]	2001-2011	Spring (May)	Winter (Feb)
	Israel [12]	2001-2011	Spring	Fall
Europe	Portugal [13]	2000-2010	Spring (Mar)	Winter (Dec)
	Spain [14]	1996-2004	Summer	Fall-winter
	Netherlands [15]	1993-2008	Spring	Winter
	UK [16]	1983-1992	Summer	Winter
Others	USA [17]	1993-2008	Spring (Mar)	Fall (Nov)
	Australia [18]	2002-2011	Fall (Oct)	Summer (Jun)
	Cameroon [19]	2002-2004	Winter (rainy season)	Summer (dry season)

**Table 2.** Seasonal number and crude rates of tuberculosis notifications, 2006-2016

Year	Spring	Summer	Fall	Winter
2006	9,041 (18.5)	9,344 (19.1)	8,484 (17.4)	8,492 (17.4)
2007	9,186 (18.7)	9,584 (19.5)	8,005 (16.3)	7,935 (16.2)
2008	8,868 (17.9)	9,184 (18.6)	8,284 (16.8)	7,821 (15.8)
2009	9,675 (19.5)	10,276 (20.7)	8,313 (16.7)	7,581 (15.3)
2010	9,560 (19.2)	10,367 (20.8)	8,655 (17.4)	7,723 (15.5)
2011	10,516 (21.0)	11,262 (22.5)	9,639 (19.2)	8,140 (16.2)
2012	10,315 (20.5)	11,077 (22.0)	9,161 (18.2)	8,992 (17.9)
2013	9,724 (19.2)	9,561 (18.9)	8,327 (16.5)	8,477 (16.8)
2014	9,438 (18.6)	9,124 (18.0)	8,172 (16.1)	8,135 (16.0)
2015	8,619 (16.9)	8,154 (16.0)	7,794 (15.3)	7,614 (14.9)
2016	8,321 (16.3)	8,371 (16.4)	7,229 (14.1)	6,971 (13.6)
Total	103,263	106,304	92,063	87,881

Values are presented as number (crude rate). Unit: cases (per 100,000 population).

**Table 3.** Monthly mean number and crude rate (CR) of tuberculosis notifications, 2006-2016

Month	Mean cases	Range		CR
		Minimun	Maximum	
Jan	2,623	2,190	3,038	5.2
Feb	2,559	2,138	3,145	5.1
Mar	3,030	2,781	3,467	6.1
Apr	3,181	2,711	3,529	6.4
May	3,176	2,713	3,649	6.3
Jun	3,210	2,777	3,768	6.4
Jul	3,346	2,774	3,889	6.7
Aug	3,109	2,577	3,825	6.2
Sep	2,792	2,321	3,242	5.6
Oct	2,813	2,450	3,147	5.6
Nov	2,765	2,458	3,330	5.5
Dec	2,807	2,409	3,291	5.6

Unit: cases (per 100,000 population).

**Table 4.** Seasonal indices by seasonal decomposition method for tuberculosis notifications in Korea

Season	2006-2016	2006-2012	2013-2016
Spring	508.140	532.164	469.568
Summer	813.315	1,108.955	318.068
Fall	-474.069	-507.491	-440.703
Winter	-847.385	-1,133.628	-346.932

## SUPPLEMENTARY MATERIALS

Supplementary Material: Korean version is available at http://www.e-epih.org/.

## References

[1] Korea Centers for Disease Control and Prevention (2017). Infectious diseases surveillance yearbook. http://www.cdc.go.kr/npt/biz/npp/portal/nppPblctDtaMain.do?pblctDtaSeAt=1.

[2] Korea Centers for Disease Control and Prevention (2018). National guideline for tuberculosis control. http://www.cdc.go.kr/CDC/together/CdcKrTogether0302.jsp?menuIds=HOME006-MNU2804-MNU3027-MNU2979&fid=10713&q_type=title&q_value=%EA%B2%B0%ED%95%B5&cid=138005&pageNum=.

[3] Lew WJ (2000). Tuberculosis surveillance system in Korea. Tuberc Respir Dis.

[4] Nnoaham KE, Clarke A (2008). Low serum vitamin D levels and tuberculosis: a systematic review and meta-analysis. Int J Epidemiol.

[5] Nagayama N, Ohmori M (2006). Seasonality in various forms of tuberculosis. Int J Tuberc Lung Dis.

[6] Wubuli A, Li Y, Xue F, Yao X, Upur H, Wushouer Q (2017). Seasonality of active tuberculosis notification from 2005 to 2014 in Xinjiang, China. PLoS One.

[7] Leung CC, Yew WW, Chan TY, Tam CM, Chan CY, Chan CK (2005). Seasonal pattern of tuberculosis in Hong Kong. Int J Epidemiol.

[8] Thorpe LE, Frieden TR, Laserson KF, Wells C, Khatri GR (2004). Seasonality of tuberculosis in India: is it real and what does it tell us?. Lancet.

[9] Khaliq A, Batool SA, Chaudhry MN (2015). Seasonality and trend analysis of tuberculosis in Lahore, Pakistan from 2006 to 2013. J Epidemiol Glob Health.

[10] Akhtar S, Mohammad HG (2008). Seasonality in pulmonary tuberculosis among migrant workers entering Kuwait. BMC Infect Dis.

[11] Moosazadeh M, Khanjani N, Bahrampour A (2013). Seasonality and temporal variations of tuberculosis in the North of Iran. Tanaffos.

[12] Margalit I, Block C, Mor Z (2016). Seasonality of tuberculosis in Israel, 2001-2011. Int J Tuberc Lung Dis.

[13] Bras AL, Gomes D, Filipe PA, de Sousa B, Nunes C (2014). Trends, seasonality and forecasts of pulmonary tuberculosis in Portugal. Int J Tuberc Lung Dis.

[14] Luquero FJ, Sanchez-Padilla E, Simon-Soria F, Eiros JM, Golub JE (2008). Trend and seasonality of tuberculosis in Spain, 1996-2004. Int J Tuberc Lung Dis.

[15] Korthals Altes H, Kremer K, Erkens C, van Soolingen D, Wallinga J (2012). Tuberculosis seasonality in the Netherlands differs between natives and non-natives: a role for vitamin D deficiency?. Int J Tuberc Lung Dis.

[16] Douglas AS, Strachan DP, Maxwell JD (1996). Seasonality of tuberculosis: the reverse of other respiratory diseases in the UK. Thorax.

[17] Willis MD, Winston CA, Heilig CM, Cain KP, Walter ND, Mac Kenzie WR (2012). Seasonality of tuberculosis in the United States, 1993- 2008. Clin Infect Dis.

[18] Maclachlan JH, Lavender CJ, Cowie BC (2012). Effect of latitude on seasonality of tuberculosis, Australia, 2002-2011. Emerg Infect Dis.

[19] Ane-Anyangwe IN, Akenji TN, Mbacham WF, Penlap VN, Titanji VP (2006). Seasonal variation and prevalence of tuberculosis among health seekers in the South Western Cameroon. East Afr Med J.

[20] Koh GC, Hawthorne G, Turner AM, Kunst H, Dedicoat M (2013). Tuberculosis incidence correlates with sunshine: an ecological 28- year time series study. PLoS One.

[21] Facchini L, Venturini E, Galli L, de Martino M, Chiappini E (2015). Vitamin D and tuberculosis: a review on a hot topic. J Chemother.

[22] Jang HB, Lee HJ, Park JY, Kang JH, Song J (2013). Association between serum vitamin d and metabolic risk factors in Korean schoolgirls. Osong Public Health Res Perspect.

[23] Lee YA, Kim HY, Hong H, Kim JY, Kwon HJ, Shin CH (2014). Risk factors for low vitamin D status in Korean adolescents: the Korea National Health and Nutrition Examination Survey (KNHANES) 2008-2009. Public Health Nutr.

[24] Cleveland WP, Tiao GC (1976). Decomposition of seasonal time series: a model for the census X-11 program. J Am Stat Asso.

[25] IBM IBM SPSS forecasting 22. http://www.sussex.ac.uk/its/pdfs/SPSS_Forecasting_22.pdf.

[26] Tedijanto C, Hermans S, Cobelens F, Wood R, Andrews JR (2018). Drivers of seasonal variation in tuberculosis incidence: insights from a systematic review and mathematical model. Epidemiology.

[27] Fares A (2011). Seasonality of tuberculosis. J Glob Infect Dis.

[28] Balcells ME, García P, Tiznado C, Villarroel L, Scioscia N, Carvajal C (2017). Association of vitamin D deficiency, season of the year, and latent tuberculosis infection among household contacts. PLoS One.

[29] Morbey RA, Elliot AJ, Harcourt S, Smith S, de Lusignan S, Pebody R (2018). Estimating the burden on general practitioner services in England from increases in respiratory disease associated with seasonal respiratory pathogen activity. Epidemiol Infect.

[30] Zürcher K, Zwahlen M, Ballif M, Rieder HL, Egger M, Fenner L (2016). Influenza pandemics and tuberculosis mortality in 1889 and 1918: analysis of historical data from Switzerland. PLoS One.

[31] Noh JY, Lee J, Choi WS, Song JY, Seo YB, Kim IS (2013). Concurrent tuberculosis and influenza, South Korea. Emerg Infect Dis.

[32] Redford PS, Mayer-Barber KD, McNab FW, Stavropoulos E, Wack A, Sher A (2014). Influenza A virus impairs control of Mycobacterium tuberculosis coinfection through a type I interferon receptor-dependent pathway. J Infect Dis.

[33] Dangor Z, Izu A, Moore DP, Nunes MC, Solomon F, Beylis N (2014). Temporal association in hospitalizations for tuberculosis, invasive pneumococcal disease and influenza virus illness in South African children. PLoS One.

[34] Smith I (2003). Mycobacterium tuberculosis pathogenesis and molecular determinants of virulence. Clin Microbiol Rev.

[35] Fisman DN (2007). Seasonality of infectious diseases. Annu Rev Public Health.

