# Supplementary material for: Seasonality of tuberculosis in the Republic of Korea, 2006-2016
Source: Epidemiol Health. 2018 Oct 20;40:e2018051. doi: 10.4178/epih.e2018051 (PMC6288684; doi:10.4178/epih.e2018051)
Supplement: Supplementary file 1 [file epih-40-e2018051-supplementary.pdf]

Volume: 40, Article ID: e2018051, 6 pages

https://doi.org/10.4178/epih.e2018051

<Original Article>

국내 결핵 발생의 계절성, 2006-2016

Seasonality of Tuberculosis in the Republic of Korea, 2006-2016

김은희[1)], 배종면[1)]

1) 제주대학교 의학전문대학원 예방의학교실

Eun Hee Kim[1)], Jong-Myon Bae[1)]

1) Department of Preventive Medicine, Jeju National University School of Medicine, Jejudo, Korea

2)

Corresponding authors: Jong-Myon Bae

jmbae@jejunu.ac.kr

Volume: 40, Article ID: e2018051, 6 pages

https://doi.org/10.4178/epih.e2018051

Abstract

Objectives: While the seasonality of notified tuberculosis has been identified in several populations, there is not a descriptive epidemiological study on the seasonality of tuberculosis in Korea. This study aimed to evaluate the seasonality of tuberculosis in Korea from 2006 to 2016.

Methods: Data regarding notified cases of tuberculosis by year and month was obtained from the Infectious Diseases Surveillance Yearbook, 2017 published by the Korea Centers for Disease Control and Prevention. Seasonal decomposition was conducted using the method of structural model of time series analysis with simple moving averages.

Results: While the trough season was winter from 2006 to 2016, the peak season was summer between 2006 and 2012, but shifted to spring between 2013 and 2016.

Conclusion: Notified tuberculosis in Korea also showed seasonality. It is necessary to evaluate factors related to the seasonality of tuberculosis for controlling tuberculosis.

Key Words: Tuberculosis, Periodicity, Disease Management, Vitamin D, Prevention

Volume: 40, Article ID: e2018051, 6 pages

https://doi.org/10.4178/epih.e2018051

서론

우리나라의 결핵 발생률은 OECD 국가 중 1위이며, 국내에서도 전수감시 감염병 중 수두 다음으로 발생 건수가 많은 감염병이다 [1]. 정부는 결핵 퇴치를 위해 1962년부터 전국 보건소 중심의 국가결핵관리체계를 구축하여 실태조사를 실시하여 왔고 [2], 2000년 이후 의료기관 중심의 웹 기반 신고시스템을 도입하여 보다 체계적인 결핵 관리를 시작하였다 [3]. 이렇게 수집된 데이터를 국가결핵관리정책 기획, 실행 및 평가를 위해 질병관리본부에서 결핵 감시 연보로 발행하고 있다 [1]. 그러나 2018년 현재 결핵 발생률은 여전히 높다는 점에서, 결핵 관리를 위한 전략 수립 등에 활용하기 위한 국내 결핵환자의 발생에 대한 역학적 특성을 밝히는 기술역학 연구 (descriptive epidemiological study)를 적극 수행할 필요가 있다.

결핵 발생에 있어 대표적인 기술역학적 특성은 계절성이 있다는 점이다 [4]. 관련한 문헌들을 정리해 볼 때, 전 세계적으로 봄 또는 여름에 결핵환자 발생이 많고, 가을과 겨울에 낮다고 보고하고 있다 [5-19] (Table 1). 이처럼 결핵 보고체계가 상이한 여러 나라에서 같은 시기의 계절성을 보인다는 점에서 가장 설득력을 가진 가설은 계절별 일조량에 따른 비타민D 농도의 영향이다 [20]. 이에, 비타민D 부족과 결핵 발생 위험간의 관련성이 있음을 가장 근거수준이 높은 체계적 고찰을 통해 보고하였다 [21,22].

한편, 국내 12-18세 청소년들에 있어 혈중 비타민D (25(OH)D) 농도를 조사한 결과, 겨울철에는 20 ng/mL 미만의 결핍상태가 대상자의 90.5%이었다 [23]. 그렇다면, 국내 결핵 발생에도 계절성이 있을 것으로 예상해 볼 수 있겠다. 그러나 2018년 9월 현재까지 국내 결핵발생의 계절성을 알아본 기술역학연구를 찾을 수 없다는 점에서, 국내 결핵 발생의 계절성 여부를 알아보는 것이 본 연구의 목적이다.

Volume: 40, Article ID: e2018051, 6 pages

https://doi.org/10.4178/epih.e2018051

대상 및 방법

결핵의 계절성 분석을 위해 필요한 연도별 월별 환자발생 신고 수는, 질병관리본부에서 발행한 2017 감염병 감시연보에서 확보하였다 [1]. 해당 자료원은 결핵 또는 결핵 의사환자로 전국 보건소 및 병의원에서 결핵정보통합관리시스템을 통해 신환자(초치료자)로 신고된 통계 정보에 따라 제시된 것이다. 따라서 재치료자와 과거치료여부가 불명확한 환자는 포함되지 않았다. 결핵환자는 결핵균이 인체 내에 침입하여 임상적 특징이 나타나는 자로서 결핵균 검사에서 양성으로 확인된 자이며, 결핵의사환자는 임상적, 방사선학적 또는 조직학적 소견상 결핵에 해당하지만 결핵균 검사에서 양성으로 확인되지 아니한 자로서 둘 다 신고대상이다.

질병관리본부는 2000년부터 웹 기반 결핵환자 신고체계를 갖추기 시작하여, 2001년부터 연도별 월별 환자 발생 신고자 통계가 제공되고 있다 [2]. 그러나 2005년 9월에 5,284건이 등록되었는데, 같은 월의 2004년도 2,301건과 2006년도 2,860건과 비교할 때 신고건수의 변동이 있었다 [1]. 분석할 통계 자료의 안정성을 위해 2006년 1월 1일부터 2016년 12월 31일까지의 10년간 월별 신고 수를 확보하였다. 계절성을 보기 위하여 봄 (Q1, 3~5월), 여름 (Q2, 6~8월), 가을 (Q3, 9~11월), 겨울 (Q4, 12~2월) 각각 3개월씩 묶어 계절을 나타내는 변수를 만들었다. 또한, 건수만으로는 성별, 연령별 정보를 알 수 없기에 연도별 계절별 신고

Volume: 40, Article ID: e2018051, 6 pages

https://doi.org/10.4178/epih.e2018051

율은 각 연도별 주민등록연앙인구를 적용한 조발생률 (crude rate)로 대치하였다.

계절성 확인을 위해 시계열 분석 중 계절 분해 기법 (seasonal decomposition)을 사용하여 계절지수 (seasonal index)를 산출하였다 [24]. 계절 분해 시계열 모형으로는 승법모형 (multiplicative approaches)과 가법모형 (additive approaches)이 있다. 승법모형은 결과 변수가 추세요인 (trend), 순환요인 (circular), 계절요인 (seasonal), 불규칙 요인 (error)의 곱으로 설명될 수 있다는 것이고, 가법모형은 4가지 요인의 합으로 설명될 수 있다는 것이다. 두 가지의 모형 중 시계열의 증가에도 계절진동이 일정하면 가법계절변동, 시계열의 증감에 따라 계절진동의 크기가 증가하거나 감소하면 승법계절변동을 선택한다 [25]. 본 연구 데이터는 시간의 흐름에 따라 계절진동의 크기가 대체로 일정하므로 가법모형을 적용하였다. 또한, 계절변동 조정을 위해 기간별 데이터의 너비가 주기와 같고, 모든 시점에서 동일하게 가중되도록 단순이동평균법 (simple moving averages method)을 적용하였다. 통계 분석 프로그램은 SPSS PASW STATISTICS 18.0 (IBM Corp., Armonk, NY, USA)을 사용하였다. 본 연구는 개인정보를 알 수 없는 자료를 사용하였기에, IRB 심의 면제를 받았다.

결과

2006년 1월 1일부터 2016년 12월 31일까지 전국에서 총 389,511건의 결핵 신고가 보고되었다. 이 중 계절별로는 여름이 106,304건으로 가장 높았고, 겨울이 87,881건으로 가장 낮았다 (Table 2). 월별로는 7월의 평균 신고수가 가장 높고, 2월이 가장 낮았다 (Table 3).

연도별 계절별 신고수 추이를 보았을 때 2006년부터 2012년까지는 여름이 가장 높고 겨울이 가장 낮은 경향을 보이다가, 2013년부터 봄이 가장 높은 추세로 변화하였다 (Fig 1). Fig 2는 2006년부터 2016년까지의 결핵 신고수의 계절 분해 결과이다. 계절요인을 제거한 계절 수정 계열의 고점이 추세 순환 계열을 상회하는 것으로 보아, 계절 변화에 따라 발생 신고율이 민감하게 변하는 것을 알 수 있다.

또한 Table 4를 통해 전체적인 신고수의 감소와 더불어 계절에 따른 추세 변화가 있음을 확인하였다. Table 4의 계절 지수를 보면 여름 813.315, 봄 508.140, 가을 –474.069, 겨울 –847.385 순으로 나타났다. 그러나 2013년부터의 추세 변화에 따라 2006~2012년, 2013~2016년으로 나누어 분석한 결과, 2006~2012년은 동일한 순서로 나타났으나, 2013~2016년은 봄 469.568, 여름 318.068, 겨울 –346.932, 가을 –440.703 순으로 변하였다.

Volume: 40, Article ID: e2018051, 6 pages

https://doi.org/10.4178/epih.e2018051

토론

이상의 결과들을 통해 우리나라의 2006~2016년도 결핵 신고에서 여름에 가장 높고 겨울에 가장 낮은 계절성을 갖는다는 것을 확인하였다. 최근 결핵 발생의 계절성에 대한 체계적 고찰 연구 결과 [26]에서 57개의 연구 중 49개의 연구에서 봄 또는 여름이 결핵 발생이 높고, 가을 또는 겨울이 결핵 발생이 낮다는 것과 같은 맥락을 갖는다.

결핵 발생의 계절적 변화 요인으로는 일조량, 기온 변화, 실내활동, 면역력의 변화 등이 제시되고 있지만 위도 또는 계절에 따른 일조량의 영향을 받는다는 가설이 가장 유력하다 [27]. 남반구의 나라에서는 북반구 나라와 반대의 계절성을 결과가 나온다는 점이 이를 뒷받침한다 [26]. 또한 겨울에 실내 활동이 많아지면서 밀집 생활을 하게 되고, 일조량이 줄어들면서 비타민D의 결핍으로 면역력이 감소하여 감염에 대한 감수성을 높이게 되는 요인들의 복합적인 연관성을 유추해 볼 수 있다 [28].

그러나 겨울에 발생이 증가하는 대부분의 호흡기계 감염병의 특성과는 다르게 결핵은 봄과 여름에 신고가 증가하는 이유들로는 다음 두 가지가 언급되고 있다 [29]. 첫째로 겨울에 발생한 호흡기계 질병이 결핵을 유발하는 것이다. 남아프리카 공화국에서 수행한 인플루엔자, 침습성 폐렴구균 질환, 결핵의 계절성 연구에서 인플루엔자의 발생이 최고조에 이른 후 약 3~4개월 뒤 침습성 폐렴구균 질환과 결핵 발생이 최고조에 이르는 것을 확인했다

[30-33]. 둘째는 결핵균의 질병 진행 기간이다. 결핵균은 체내에 침입한 후 증상 발현까지 장시간이 걸린다. 결핵균이 폐포에 침입한 후 투베르쿨린 반응으로 전환되기까지 3~8주가 걸리고, 증상발현까지 약 3개월이 소요되며 이 기간은 개인별 차이에 의해 2년까지 지연될 수 있다 [34]. 이러한 이유로 결핵의 발병 특성을 반영하여 결핵 신고일을 기준으로 분석한 본 연구의 결과를 해석해 볼 때, 겨울에서 봄에 이르는 시기에 감염이 되어 봄과 여름에 신고되었을 것으로 유추할 수 있다.

본 연구 결과에서 주목할 점은 2013년부터 신고수가 감소하면서 발생의 최고점이 여름에서 봄으로 이동했다는 것이다. 다만 신고 발생의 최고점에서만 이동변화가 있었고 겨울이 여전히 가장 낮은 추세는 변함이 없다는 점에서, 결핵의 역학적 발생 특성의 변화보다는 국가 정책이나 신고 시스템의 변화에 의한 것으로 유추할 수 있다. 다시 말해서, 국가결핵 관리 정책의 강화로 인하여 적극적으로 결핵 환자를 발견함으로써 발생에서부터 신고까지의 기간이 줄어든 것으로 해석해 볼 수 있겠다. 보건당국은 2013년에 수립한 '제1기 결핵관리종합계획(2013~2017)'에 따라, 고위험군을 선정하고 능동적으로 검사를 진행하여 환자를 조기 발견하고 치료까지 이어질 수 있도록 환자 관리를 강화해 왔다 [2]. 이런 노력들로 인하여 2013년에 결핵 신고수의 감소와 신고기간 단축을 이끌어냈다고 유추할 수 있다.

본 연구의 한계점은 다음과 같다. 첫째, 결핵 신환자 신고 수만 이용하여 우리나라

Volume: 40, Article ID: e2018051, 6 pages

https://doi.org/10.4178/epih.e2018051

의 성별, 연령별 등의 기본적인 특성을 확인할 수 없다는 점이다. 또한 데이터에 외국인과 의사환자가 포함되어 있어 연구 대상을 구체화할 수 없었다. 그러나 일조량과 국적과는 무관하며, 매년 만명 단위의 신고 건수 크기를 고려할 때, 외국인과 의사환자들이 포함된 것이 계절성 변동에 교란을 일으키지 않을 것으로 본다. 향후 결핵환자의 인적 특성에 맞춘 기술역학연구가 추가로 필요하겠다. 둘째, 결핵의 특성상 연보통계만으로는 결핵 발생 시점을 명확히 알기 어렵다는 점이다. 결핵의 잠복기가 길어서 발생 시기를 정확히 추정하기 어렵기 때문이다. 그러나 초진 환자에 대한 연보통계라는 점을 활용하여, 결핵 신고 시점을 발생 시점으로 간주하였다. 셋째, 기술역학연구이므로 한국인 결핵신고의 계절성을 야기하는 이유들에 대한 추가 연구가 필요하다. 성별, 연령별로 계절과 실내 활동시간에 따른 비타민 D 농도 수준을 알아내고, 결핵 발생과의 연관성을 알아내는 분석역학연구가 필요하다.

결핵 발생의 계절성에 대한 이해는 효율적이고 효과적으로 결핵을 예방하기 위해 아주 중요하다 [27]. 계절별 차이 중 결핵 발생에 관여할 것으로 추정되는 요인들을 확인하여 차단하거나 보완하는 방법으로 발생이 높은 계절에 집중적인 예방대책을 수립할 수 있기 때문이다. 또한 결핵의 병인학적 지식 향상에 기여하고 [17], 감시체계를 공고히 하는데 역시 도움이 되기 때문이다 [35].

Volume: 40, Article ID: e2018051, 6 pages

https://doi.org/10.4178/epih.e2018051

참고문헌

1. KCDC. Infectious Diseases Surveillance Yearbook, 2017. [cited 2018 Sep 20]. Available from: http://www.cdc.go.kr/npt/biz/npp/portal/nppPblctDtaMain.do?pblctDtaSeAt=1.

2. KCDC. National Guideline for Tuberculosis Control, 2018. [cited 2018 Sep 20]. Available from: http://www.cdc.go.kr/CDC/together/CdcKrTogether0302.jsp?menuIds=HOME006-MNU2804-MNU3027-MNU2979&fid=10713&q_type=title&q_value=%EA%B2%B0%ED%95%B5&cid=138005&pageNum=.

3. Lew WJ. Tuberculosis surveillance system in Korea. Tuberc Respir Dis. 2000;48:298-307.

4. Nnoaham KE, Clarke A. Low serum vitamin D levels and tuberculosis: a systematic review and meta-analysis. Int J Epidemiol. 2008;37(1):113-9.

5. Nagayama N, Ohmori M. Seasonality in various forms of tuberculosis. Int J Tuberc Lung Dis. 2006;10(10):1117-22.

6. Wubuli A, Li Y, Xue F, Yao X, Upur H, Wushouer Q. Seasonality of active tuberculosis notification from 2005 to 2014 in Xinjiang, China. PLoS One. 2017;12(7):e0180226.

7. Leung CC, Yew WW, Chan TY, Tam CM, Chan CY, Chan CK, et al. Seasonal pattern of tuberculosis in Hong Kong. Int J Epidemiol. 2005;34(4):924-30.

8. Thorpe LE, Frieden TR, Laserson KF, Wells C, Khatri GR. Seasonality of tuberculosis in India: is it real and what does it tell us? Lancet. 2004;364(9445):1613-4.

9. Khaliq A, Batool SA, Chaudhry MN. Seasonality and trend analysis of tuberculosis in Lahore, Pakistan from 2006 to 2013. J Epidemiol Glob Health. 2015;5(4):397-403.

10. Akhtar S, Mohammad HG. Seasonality in pulmonary tuberculosis among migrant workers entering Kuwait. BMC Infect Dis. 2008;8:3.

11. Moosazadeh M, Khanjani N, Bahrampour A. Seasonality and temporal variations of tuberculosis in the north of iran. Tanaffos. 2013;12(4):35-41.

12. Margalit I, Block C, Mor Z. Seasonality of tuberculosis in Israel, 2001-2011. Int J Tuberc Lung

Dis. 2016;20(12):1588-1593.

13. Bras AL, Gomes D, Filipe PA, de Sousa B, Nunes C. Trends, seasonality and forecasts of pulmonary tuberculosis in Portugal. Int J Tuberc Lung Dis. 2014;18(10):1202-10.
14. Luquero FJ, Sanchez-Padilla E, Simon-Soria F, Eiros JM, Golub JE. Trend and seasonality of tuberculosis in Spain, 1996-2004. Int J Tuberc Lung Dis. 2008;12(2):221-4.
15. Korthals Altes H1, Kremer K, Erkens C, van Soolingen D, Wallinga J. Tuberculosis seasonality in the Netherlands differs between natives and non-natives: a role for vitamin D deficiency? Int J Tuberc Lung Dis. 2012;16(5):639-44.
16. Douglas AS, Strachan DP, Maxwell JD. Seasonality of tuberculosis: the reverse of other respiratory diseases in the UK. Thorax. 1996;51(9):944-6.
17. Willis MD, Winston CA, Heilig CM, Cain KP, Walter ND, Mac Kenzie WR. Seasonality of tuberculosis in the United States, 1993-2008. Clin Infect Dis. 2012;54(11):1553-60.
18. Maclachlan JH, Lavender CJ, Cowie BC. Effect of latitude on seasonality of tuberculosis, Australia, 2002-2011. Emerg Infect Dis. 2012;18(11):1879-81.
19. Ane-Anyangwe IN, Akenji TN, Mbacham WF, Penlap VN, Titanji VP. Seasonal variation and prevalence of tuberculosis among health seekers in the South Western Cameroon. East Afr Med J. 2006;83(11):588-95.
20. Koh GC, Hawthorne G, Turner AM, Kunst H, Dedicoat M. Tuberculosis incidence correlates with sunshine: an ecological 28-year time series study. PLoS One. 2013;8(3):e57752.
21. Facchini L, Venturini E, Galli L, de Martino M, Chiappini E. Vitamin D and tuberculosis: a review on a hot topic. J Chemother. 2015;27(3):128-38.
22. Jang HB, Lee HJ, Park JY, Kang JH, Song J. Association between serum vitamin d and metabolic risk factors in korean schoolgirls. Osong Public Health Res Perspect. 2013;4(4):179-86.
23. Lee YA, Kim HY, Hong H, Kim JY, Kwon HJ, Shin CH, et al. Risk factors for low vitamin D status in Korean adolescents: the Korea National Health and Nutrition Examination Survey

(KNHANES) 2008-2009. Public Health Nutr. 2014;17(4):764-71.

24. Cleveland WP, Tiao GC. Decomposition of seasonal time series: a model for the census X-11 program. J Am Stat Assoc. 1976:71(355):581-7.

25. IBM. SPSS Forecasting 22. [cited 2018 Sep 20]. Available from: http://www.sussex.ac.uk/its/pdfs/SPSS_Forecasting_22.pdf.

26. Tedijanto C, Hermans S, Cobelens F, Wood R, Andrews JR. Drivers of seasonal variation in tuberculosis incidence: insights from a systematic review and mathematical model. Epidemiology. 2018.

27. Fares A. Seasonality of tuberculosis. J Glob Infect Dis. 2011;3(1):46-55.

28. Balcells ME, García P, Tiznado C, Villarroel L, Scioscia N, Carvajal C, et al. Association of vitamin D deficiency, season of the year, and latent tuberculosis infection among household contacts. PLoS One. 2017;12(4):e0175400.

29. Morbey RA, Elliot AJ, Harcourt S, Smith S, de Lusignan S, Pebody R, et al. Estimating the burden on general practitioner services in England from increases in respiratory disease associated with seasonal respiratory pathogen activity. Epidemiol Infect. 2018;146(11):1389-1396.

30. Zürcher K, Zwaheln M, Ballif M, Rieder HL, Egger M, Fenner L. Influenza pandemics and tuberculosis mortality in 1889 and 1919: analysis of historical data from Switzerland. PLoS One. 2016;11(10):e0162575

31. Noh JY, Lee J, Choi WS, Song JY, Seo YB, Kim IS, Cheong HJ, Kim WJ. Concurrent tuberculosis and influenza, South Korea. Emerg Infect Dis. 2013;19(1):165-167.

32. Redford PS, Mayer-Barber KD, McNab FW, Stavropoulos E, Wack A, Sher A, et al. Influenza A virus impairs control of Mycobacterium tuberculosis coinfection through a type I interferon receptor-dependent pathway. J Infect Dis. 2014;209(2):270-274.

33. Dangor Z, Izu A, Moore DP, Nunes MC, Solomon F, Beylis N, et al. Temporal association in

Volume: 40, Article ID: e2018051, 6 pages

https://doi.org/10.4178/epih.e2018051

hospitalizations for tuberculosis, invasive pneumococcal disease and influenza virus illness in South African children. PLoS One. 2014;9(3):e91464.

34. Smith I. Mycobacterium tuberculosis pathogenesis and molecular determinants of virulence. Clin Microbiol Rev. 2003;16(3):463-96.

35. Fisman DN. Seasonality of infectious diseases. Annu Rev Public Health. 2007;28:127-43.
